# Prevalence of SARS-CoV-2 in six districts in Zambia in July, 2020: a cross-sectional cluster sample survey

**DOI:** 10.1016/S2214-109X(21)00053-X

**Published:** 2021-03-09

**Authors:** Lloyd B Mulenga, Jonas Z Hines, Sombo Fwoloshi, Lameck Chirwa, Mpanji Siwingwa, Samuel Yingst, Adam Wolkon, Danielle T Barradas, Jennifer Favaloro, James E Zulu, Dabwitso Banda, Kotey I Nikoi, Davies Kampamba, Ngawo Banda, Batista Chilopa, Brave Hanunka, Thomas L Stevens, Aaron Shibemba, Consity Mwale, Suilanji Sivile, Khozya D Zyambo, Alex Makupe, Muzala Kapina, Aggrey Mweemba, Nyambe Sinyange, Nathan Kapata, Paul M Zulu, Duncan Chanda, Francis Mupeta, Chitalu Chilufya, Victor Mukonka, Simon Agolory, Kennedy Malama

**Affiliations:** **Zambia Ministry of Health, Lusaka, Zambia** (L B Mulenga PhD, S Fwoloshi MMed, A Shibemba MMed, S Sivile MMed, K D Zyambo MMed, A Makupe MMed, A Mweemba MMed, C Chilufya MBChB, K Malama MBChB); **University Teaching Hospital, Lusaka, Zambia** (L B Mulenga, S Fwoloshi, L Chirwa MPH, M Siwingwa MPH, K I Nikoi BSc, D Kampamba MSc, A Shibemba, S Sivile, K D Zyambo, A Makupe, A Mweemba, D Chanda MMed, F Mupeta MMed); **Levy Mwanawasa Medical University, Lusaka, Zambia** (L B Mulenga, S Fwoloshi, A Shibemba, S Sivile, K D Zyambo, A Makupe, A Mweemba, F Mupeta, C Mwale); **Vanderbilt Medical University, Nashville, TN, USA** (L B Mulenga); **School of Medicine, University of Zambia, Lusaka, Zambia** (L B Mulenga, S Fwoloshi, A Shibemba, C Mwale MMed, S Sivile, A Makupe, A Mweemba, D Chanda, F Mupeta); **Centers for Disease Control and Prevention, Lusaka, Zambia** (J Z Hines MD, S Yingst PhD, A Wolkon MPH, D T Barradas PhD, B Hanunka MSc, T L Stevens Jr DrPH, S Agolory MD); **Centers for Disease Control and Prevention, Atlanta, GA, USA** (J Favaloro MS); **Zambia Field Epidemiology Training Program, Lusaka, Zambia** (J E Zulu MBChB, D Banda MBChB, N Sinyange MSc); **Zambia National Public Health Institute, Lusaka, Zambia** (J E Zulu, D Banda, M Kapina MBChB, N Sinyange, N Kapata PhD, P M Zulu MD, V Mukonka PhD); **Pan-African Network for Rapid Research, Response, Relief and Preparedness for Infectious Diseases Epidemics, Amsterdam, Netherlands** (N Kapata); **Zambia Statistics Agency, Lusaka, Zambia** (N Banda BAS, B Chilopa BSc); **Lusaka Provincial Health Office, Lusaka, Zambia** (C Mwale)

## Abstract

**Background:**

Between March and December, 2020, more than 20 000 laboratory-confirmed cases of SARS-CoV-2 infection were reported in Zambia. However, the number of SARS-CoV-2 infections is likely to be higher than the confirmed case counts because many infected people have mild or no symptoms, and limitations exist with regard to testing capacity and surveillance systems in Zambia. We aimed to estimate SARS-CoV-2 prevalence in six districts of Zambia in July, 2020, using a population-based household survey.

**Methods:**

Between July 4 and July 27, 2020, we did a cross-sectional cluster-sample survey of households in six districts of Zambia. Within each district, 16 standardised enumeration areas were randomly selected as primary sampling units using probability proportional to size. 20 households from each standardised enumeration area were selected using simple random sampling. All members of selected households were eligible to participate. Consenting participants completed a questionnaire and were tested for SARS-CoV-2 infection using real-time PCR (rtPCR) and anti-SARS-CoV-2 antibodies using ELISA. Prevalence estimates, adjusted for the survey design, were calculated for each diagnostic test separately, and combined. We applied the prevalence estimates to census population projections for each district to derive the estimated number of SARS-CoV-2 infections.

**Findings:**

Overall, 4258 people from 1866 households participated in the study. The median age of participants was 18·2 years (IQR 7·7–31·4) and 50·6% of participants were female. SARS-CoV-2 prevalence for the combined measure was 10·6% (95% CI 7·3–13·9). The rtPCR-positive prevalence was 7·6% (4·7–10·6) and ELISA-positive prevalence was 2·1% (1·1–3·1). An estimated 454 708 SARS-CoV-2 infections (95% CI 312 705–596 713) occurred in the six districts between March and July, 2020, compared with 4917 laboratory-confirmed cases reported in official statistics from the Zambia National Public Health Institute.

**Interpretation:**

The estimated number of SARS-CoV-2 infections was much higher than the number of reported cases in six districts in Zambia. The high rtPCR-positive SARS-CoV-2 prevalence was consistent with observed community transmission during the study period. The low ELISA-positive SARS-CoV-2 prevalence might be associated with mitigation measures instituted after initial cases were reported in March, 2020. Zambia should monitor patterns of SARS-CoV-2 prevalence and promote measures that can reduce transmission.

## Introduction

In Zambia, the first cases of COVID-19—caused by SARS-CoV-2—were identified on March 18, 2020.^1^ The Zambian Government acted swiftly to control the spread of SARS-CoV-2, initiating a whole-of-government response, restricting travel into the country, closing public gathering spaces (eg, restaurants, bars, churches), and invoking the Public Health Act to expand authority of the Zambian Government agencies.^2^ From the outset, contact tracing teams rapidly responded to newly reported cases. With the exception of a localised outbreak in Nakonde District in May, 2020, the number of positive cases remained sporadic until June, 2020 (appendix p 2). The number of laboratory-confirmed cases rapidly increased in July, 2020, coinciding with a gradual relaxation of physical distancing measures in May and June, 2020. According to the Zambia National Public Health Institute (ZNPHI), as of Feb 18, 2021, 72 467 confirmed COVID-19 cases had been identified from 1 038 573 tests in Zambia.

The true extent of SARS-CoV-2 infections in Zambia is likely to be greater than reported. Many people with SARS-CoV-2 infection do not come to the attention of the health system because a large proportion have asymptomatic infections and most symptomatic people have only a mild clinical illness.^3,4^ COVID-19 symptoms overlap with those of other common upper respiratory tract infections that are usually self-limited.^5^ Furthermore, limited testing capacity and surveillance system gaps are likely to have contributed to under-ascertainment of SARS-CoV-2 infections in Zambia. Although testing criteria were rapidly expanded in the country to capture cases without an international travel history,^1^ this strategy was implemented incompletely throughout the country, partly due to low rates of testing as a result of poor availability of testing supplies and reagents (approximately 0·25 tests per 1000 people per week between March and July, 2020).^6^ This situation is similar to other parts of the world; serological studies from the USA, Spain, and Brazil identified an order of magnitude or more difference between laboratory-confirmed case counts and community infections.^7–10^

Little information is available about the prevalence of SARS-CoV-2 in Africa. In a small community-based study done in April, 2020, in Addis Ababa, Ethiopia, seroprevalence was estimated to be 8·8%, whereas a large study in Maputo and Quelimane, Mozambique, estimated seroprevalence was approximately 2–4% in August, 2020.^11–13^ In Niger State, Nigeria, seroprevalence among a small sample of randomly selected individuals was 25·4% in late June, 2020.^14^ In Cape Town, South Africa, seroprevalence among several selected groups was 44·6% during the downslope of the first wave.^15,16^ In May and June, 2020, SARS-CoV-2 seroprevalence was 12·3% among health-care workers in Blantyre, Malawi.^17^ Among blood donors in Kenya, SARS-CoV-2 seroprevalence was 5·2% from April to June, 2020.^18^ Modelled estimates from Kenya suggest more widespread disease in the country, with lower severity than that observed in other regions of the world.^19^ Differences in population demographics (ie, young age structure of populations) and disease epidemiology (ie, high prevalence of infectious diseases such as HIV, tuberculosis, and malaria) in Africa compared with other heavily affected areas might affect SARS-CoV-2 epidemiology. Representative studies are needed to understand the epidemiology of SARS-CoV-2 in Africa to inform national public health responses. We aimed to estimate SARS-CoV-2 prevalence in six districts of Zambia in July, 2020, using a population-based household survey.

## Methods

### Study design and study population

We did a multistage, cross-sectional cluster-sample survey of households in six districts of Zambia (Kabwe, Livingstone, Lusaka, Nakonde, Ndola, and Solwezi) between July 4 and July 27, 2020 (appendix p 3). We selected the six districts on the basis of the high number of cases reported in these districts by ZNPHI (80% of laboratory-confirmed cases in Zambia between March and June, 2020) and because they are highly populated areas, transit corridors, or points-of-entry to Zambia. The combined population of the districts was 4 290 107 people, which accounts for a quarter of the Zambian population.^20^

Within each district, 16 standardised enumeration areas were randomly selected as primary sampling units using probability proportional to size. All households within each standardised enumeration area were listed and 20 households from each standardised enumeration area were selected using simple random sampling. All individuals (of any age) who had slept in the house the night before the survey was done were eligible for participation in the survey.

Written informed consent was obtained for adults (aged ≥18 years) and emancipated minors, parental consent was obtained for participants aged 17 years and younger, and assent was obtained for participants aged 7–17 years, before the study. The study was approved by the Zambia National Health Research Authority and the University of Zambia Biomedical Research Ethics Committee. The study was reviewed in accordance with Centers for Disease Control and Prevention (CDC) human research protection procedures and was determined to be research, but CDC investigators did not interact with any individuals or have access to identifiable data or specimens for research purposes. The study methods were aligned with those of the WHO Unity Studies.^21^

### Procedures

Participants were administered a questionnaire that included information about demographics, medical history, SARS-CoV-2 exposures, and history of recent illness on a tablet using the research electronic data capture (REDCap) application hosted by the Zambia Ministry of Health (Lusaka, Zambia). SARS-CoV-2 exposures included known contact with a laboratory-confirmed case, travel (domestic or international), usual means of transportation, health facility use in the past month, in-person attendance to work or school, and the number of visits to markets or grocery stores. Recent illness was assessed by asking if the participant had experienced any illnesses since February, 2020 (ie, before the first reported case in Zambia); if they responded affirmatively, symptomology was ascertained. All responses to the questionnaire were self-reported.

Participants were tested for SARS-CoV-2 infection by real-time PCR (rtPCR) using nasopharyngeal specimens, and for anti-SARS-CoV-2 antibodies by ELISA using plasma specimens at the University Teaching Hospital (Lusaka, Zambia) and the Centre for Infectious Disease Research in Zambia, (Lusaka, Zambia). Nasopharyngeal specimens were collected using scored swabs (Citoswab; Citotest Labware, Haimen, China). With a participant tilting their head back slightly, the swab was inserted until encountering physical resistance, rotated briefly, and withdrawn and placed into a 5 mm specimen bottle containing a viral transport medium. Blood specimens for antibody testing were collected in 500 μL edetic acid cryovial microtainer tubes using finger-prick or heel-prick (for children aged <6 months); venepuncture for blood was used as an alternative procedure in the event that finger-prick or heel-prick was unsuccessful, or according to the participant’s preference. All study specimens were transported in cooler boxes on ice to a local laboratory in each district on the same day. Blood specimens were centrifuged to separate plasma, which was transferred into a separate cryovial and stored at −20°C or below pending testing.

RNA extraction for rtPCR was done using the QIAamp Viral RNA Mini kit (Qiagen, Hilden, Germany) according to manufacturer’s instructions. The Maccura COVID-19 PCR assay (Maccura Biotechnology, Chengdu, China) was used as the primary PCR diagnostic on the QuantStudio 3 platform (ThermoFisher Scientific, Waltham, MA, USA). The algorithm for test interpretation can yield a final result of suspect if only one sample is available. Therefore, we used the publicly released CDC assay method^22^ to resolve or confirm any non-negative results and the result of the CDC assay was considered final. Primers and probes for the CDC assay were obtained from Inqaba (Johannesburg, South Africa).

The Euroimmun ELISA (PerkinElmer, Waltham, MA, USA) for anti-spike protein IgG was done in single replicate according to manufacturer’s instructions. Positive or negative results were considered final. Borderline results were re-run in duplicate and considered positive or negative if both results from the duplicate run were positive or negative; the final result was deemed borderline if both results from the duplicate were borderline or if either duplicated result was discrepant.

Positive rtPCR results were communicated to district teams for case investigation and contact tracing per national guidelines. Negative rtPCR and all ELISA results were returned to participants by study staff.

Participants could participate in the survey interview, rtPCR testing, and serological testing according to participant preference. A combined SARS-CoV-2 measure was constructed for the subset of participants who had both rtPCR and ELISA tests (appendix p 4); people with a positive rtPCR or ELISA result were considered to have had SARS-CoV-2 infection, whereas people with negative rtPCR and ELISA results were considered negative.

### Statistical analysis

SARS-CoV-2 prevalence and 95% CIs were calculated as the number of positive test results divided by the total number of tests done overall and per district and overall during the survey period. Estimates were calculated for rtPCR and ELISA separately and for the combined measure (rtPCR and ELISA). We calculated prevalence ratios (PRs) for the combined measure using Poisson regression to assess for associations between demographic and behavioural factors and for SARS-CoV-2 prevalence. The χ² test was used to assess differences in prevalence across districts. Sampling weights were calculated based on the sampling frame and non-response weights (for questionnaire and each laboratory test) were calculated at the household, standardised enumeration area, and district levels. Additionally, each set of weights was calibrated to the population estimates at the district level by age and sex. Estimates were weighted, thus raw participant numbers were not reported in the analysis. Variance estimation accounted for clustering at districts and standardised enumeration areas when calculating 95% CIs and during hypothesis testing. An intracluster correlation coefficient of 0·12 (95% CI 0·06–0·18) was calculated using ANOVA to assess the degree of household clustering of SARS-CoV-2. Analyses were done using SAS (version 9.4) and the svy package in R (version 4.0.3).

District-level estimates were applied to 2020 district-specific population projections from the Zambia Statistics Agency to estimate the total number of SARS-CoV-2 infections in each district.^20^ These numbers were compared with the total number of reported cases in each district at the end of the study (July 31, 2020) to estimate the ratio of reported cases to total SARS-CoV-2 infections in each district. Additionally, the proportion of people who reported knowing their positive SARS-CoV-2 status before testing was reported for rtPCR and ELISA tests separately. We also did a sensitivity analysis excluding 333 participants for whom epidemiological data were disassociated from laboratory results during the study.

### Role of the funding source

The funder of the study was involved in the study design, data analysis, and data interpretation, and writing of the report.

## Results

2061 households were randomly selected, for which 1866 (90·5%) heads of household agreed to participate in the study. From these households, 4258 (90·8%) of 4690 people consented to interview, and of these participants, 3742 (87·8%) provided a laboratory specimen (appendix p 4). 2990 people provided a laboratory specimen for rtPCR and 2704 people for ELISA; of these, 1952 people provided a laboratory specimen for the combined measure (both rtPCR and ELISA; appendix p 4).

50·6% of participants were female (table 1). The median age of participants was 18·2 years (IQR 7·7–31·4). 63·1% of participants resided in Lusaka District; 93·0% of participants resided in urban areas. Overall, 14·5% of participants reported having a history of a comorbid medical condition, with HIV (5·1%) and hypertension (3·8%) most common. Among people with HIV, 98·3% reported taking antiretroviral therapy (ART).

The pooled prevalence for the combined SARS-CoV-2 measure was 10·6% (95% CI 7·3–13·9) (table 2); SARS-CoV-2 prevalence varied by district from 6·0% (2·9–9·1) in Kabwe District to 14·4% (9·0–19·9) in Ndola District. No significant differences in SARS-CoV-2 prevalence were identified across districts (p=0·22, χ² test), although prevalence was higher among people who resided in urban areas than in rural areas (PR 1·08 [1·03–1·13]). The pooled rtPCR-positive prevalence was 7·6% (4·7–10·6) and ELISA-positive prevalence was 2·1% (1·1–3·1).

454708 SARS-CoV-2 infections (95% CI 312705–596713) were estimated to have occurred in the six districts between March and July, 2020, versus 4917 cases reported in official statistics. Thus one laboratory-confirmed case was reported for every 92 SARS-CoV-2 infections across the six districts (ratio of reported cases to estimated infections ranged from 1:1012 in Livingstone District to 1:21 in Nakonde District; table 3). Only 2·3% of people with positive rtPCR tests and 8·2% of people with positive ELISA test were aware of their positive status before testing.

SARS-CoV-2 pooled prevalence for the combined measure increased with age (table 4, figure). Compared with participants aged 0–9 years, prevalence was higher among individuals aged 10–19 years (PR 1·04 [95% CI 1·00–1·08]), 20–29 years (1·06 [1·02–1·10]), 30–39 years (1·10 [1·01–1·20]), and people aged 50 years and older (1·10 [1·00–1·22]). However, no association was identified between SARS-CoV-2 prevalence and sex (0·98 [0·95–1·02]). No associations were identified between SARS-CoV-2 prevalence and comorbid medical conditions (1·02 [0·96–1·08]), or HIV infection (1·07 [0·93–1·23]). Contact with a person with confirmed COVID-19 was rarely reported, but SARS-CoV-2 prevalence was lower among people who reported contact with a confirmed COVID-19 case than those with no reported contact (0·94 [0·89–0·99]). Number of market visits in the past month was associated with SARS-CoV-2 prevalence (3–5 visits *vs* 0 visits, 1·08 [1·00–1·18]). Other potential risk factors, such as travel history, usual means of transportation, and visits to health facilities were not associated with SARS-CoV-2 prevalence.

Of participants with SARS-CoV-2, 23·8% reported symptoms (appendix p 5). Among participants with symptomatic SARS-CoV-2 infections, the most common were headache (63·6%), chills (40·9%), cough (25·7%), rhinorrhoea (21·7%), and fever (16·0%).

## Discussion

In this representative study of six districts in Zambia, the prevalence of SARS-CoV-2 infection determined by rtPCR was high, corresponding with observed community-wide transmission during this study that coincided with the first wave of the COVID-19 epidemic in Zambia. Conversely, the seroprevalence of SARS-CoV-2 detected by ELISA was low, indicating that there might have been little transmission before the study period. Applying the study’s estimates to the district populations showed that the number of laboratory-confirmed cases reported in official statistics underestimated SARS-CoV-2 infections by a factor of 92. This case detection ratio of 1·1% was similar to a report from Cape Town, where an estimated 4·0% of people with COVID-19 were ascertained by the public health system.^15^ In France, an estimated 14% of symptomatic infections were detected by the public health system between May and June, 2020.^23^

The low prevalence of previous SARS-CoV-2 infection (measured by ELISA) might have resulted from the stringent physical and social distancing measures implemented by the Zambian Government after the first cases were reported. Such measures included screening and mandatory 14-day quarantine of all people entering Zambia, closing public gathering spaces, testing of anyone with symptoms of COVID-19, isolating all patients who tested positive for COVID-19 at government facilities, tracing of contacts and daily monitoring for any COVID-19 symptoms, and testing of direct contacts including the asymptomatic contacts of known COVID-19 cases. Conversely, the observed community transmission across the six districts might have resulted from relaxation of mitigation measures (ie, reopening of businesses and churches) and Zambians relaxing individual preventive measures because of a perceived low personal risk of COVID-19. The first wave of the COVID-19 epidemic in Zambia peaked in August, 2020; thus, SARS-CoV-2 transmission remained high several weeks after the study concluded. Even if all people infected with SARS-CoV-2 during the first wave developed immunity, it can be assumed most Zambians remained susceptible to SARS-CoV-2 infection on the basis of the overall prevalence observed in this study, and additional waves were expected. At the time of writing, Zambia was experiencing a second wave of SARS-CoV-2 infections that began in mid-December, 2020, which coincided with detection of the more transmissible SARS-CoV-2 501Y.V2 (B.1.351) variant, first detected in South Africa.^24^

56·5% of reported confirmed cases in Zambia to July 31, 2020, were in Lusaka District (ZNPHI). However, no significant differences in SARS-CoV-2 prevalence estimates were identified between the six districts in our study. This suggests a potential ascertainment bias resulting from a higher level of testing in Lusaka District than elsewhere in Zambia and incomplete coverage of surveillance systems. Prevalence surveys that measure the distribution of SARS-CoV-2 in the population are important tools to address this bias from uneven distribution of testing. A nationwide SARS-CoV-2 prevalence survey in Zambia is needed to assess the extent and nature of possible ascertainment bias.

Paradoxically, people reporting contact with a confirmed COVID-19 case had lower SARS-CoV-2 prevalence in this study. It is possible that individuals with known exposure to people with COVID-19 took additional individual preventive measures to avoid becoming infected. Furthermore, many people were likely being unknowingly exposed within the community considering the widespread transmission in July, 2020, in Zambia.

Data on SARS-CoV-2 infection among people with HIV are scarce. A large study from South Africa found increased mortality among people with HIV in Western Cape,^25^ whereas studies from the USA and Europe have suggested similar severity in people with and without HIV infection.^26–28^ In Cape Town, people with HIV had higher seroprevalence than women attending antenatal clinics.^15^ In an HIV clinic in Barcelona, Spain, the incidence of COVID-19 among patients was lower than that among the general population of the city.^29^ Our study was done in a country experiencing a generalised HIV epidemic, and thus a large proportion of the study population had HIV. Although the prevalence of SARS-CoV-2 was higher among people with HIV than those who were HIV negative in this study, the difference was not significant; however, this study was not powered to detect such a difference. It is unclear what effect ART, which nearly all people with HIV reported taking, had on this finding. Further studies are needed to understand the effect of HIV on SARS-CoV-2 infection severity and prevalence considering the burden of HIV in Zambia and Africa overall.

Most people with SARS-CoV-2 infection in this study were asymptomatic. Although this finding is consistent with a report from Mozambique, where 71% of residents in Maputo with serological evidence of SARS-CoV-2 infection were asymptomatic,^12^ the proportion of asymptomatic infections in this study is higher than reported elsewhere.^3,30^ Recall bias could have reduced symptom reporting; however, a high proportion of asymptomatic SARS-CoV-2 infections could help explain the paradox between the large number of SARS-CoV-2 infections estimated in this study and the relatively mild strain on hospital services observed during the first epidemic peak in Zambia compared with experiences in Europe and North America. The lower apparent severity observed might be a result of the young population in Zambia, since younger individuals are less likely to have symptoms and develop severe illness than older individuals.^31^

This study had several limitations. Although a quarter of the Zambian population reside in the six districts selected for the study, the generalisability of the findings to all 116 districts of Zambia is unknown. Furthermore, the sample was heavily weighted to Lusaka District (which has the largest population of all districts in Zambia). This study was done primarily in urban areas, but more than 50% of Zambia’s population reside in rural districts.^20^ Participants voluntarily participated in each aspect of the study (ie, interview and nasopharyngeal and blood specimen collection), and the response rate for participants who had both rtPCR and ELISA tests was low (46% of all participants), which could have biased estimates; therefore, rtPCR and ELISA prevalence estimates were also reported separately. The pooled estimate should be interpreted with caution because the districts in this study were purposefully selected. Past medical history (including HIV status) and potential exposure to SARS-CoV-2 might have been misreported. Data collection occurred during a dynamic period in the COVID-19 outbreak in Zambia, complicating interpretation of the estimates. Some individuals shed SARS-CoV-2 genetic material for weeks and some can quickly mount an antibody response; however, rtPCR positivity is likely to reflect SARS-CoV-2 infection in the past 2–3 weeks, whereas ELISA for IgG antibodies is likely to reflect past infection. The ELISA used has a reported sensitivity of about 90%, and serological cross-reactivity is an emerging area of investigation in Africa.^32,33^ Since this is an observational study, causality between reported associations cannot be determined.

Although many more SARS-CoV-2 infections have occurred than have been reported in Zambia, most Zambians remain susceptible to SARS-CoV-2 infection. Only a small proportion of people with SARS-CoV-2 infection were aware of their infection. Expanding testing capacity, including through rapid antigen testing in populations with high pretest probability of SARS-CoV-2 infection,^34^ will help rapidly detect SARS-CoV-2 infections, allowing for early isolation of infected people and timely identification of contacts, while helping to curb SARS-CoV-2 transmission in Zambia. Serial prevalence surveys will provide the Zambian Government insight with regard to the true extent of disease transmission over time and can inform vaccine strategy. Depending on the stage of the epidemic at the time of subsequent prevalence studies, the use of both rtPCR and ELISA testing should be considered because—as shown in this study—the relatively large proportion of people with rtPCR-positive SARS-CoV-2 infection would have been missed if participants had only been tested by ELISA for anti-SARS-CoV-2 antibodies. While SARS-CoV-2 continues to spread in the community, the Zambian Government should continue to aggressively promote community mitigation measures, including rapid detection and isolation of people with confirmed SARS-CoV-2 infection, identification and quarantine of people who have been in close contact with confirmed cases, universal mask wearing in public, and physical distancing measures, which have been shown to reduce SARS-CoV-2 transmission.

## Supplementary Material

Appendix

## Figures and Tables

**Figure:**
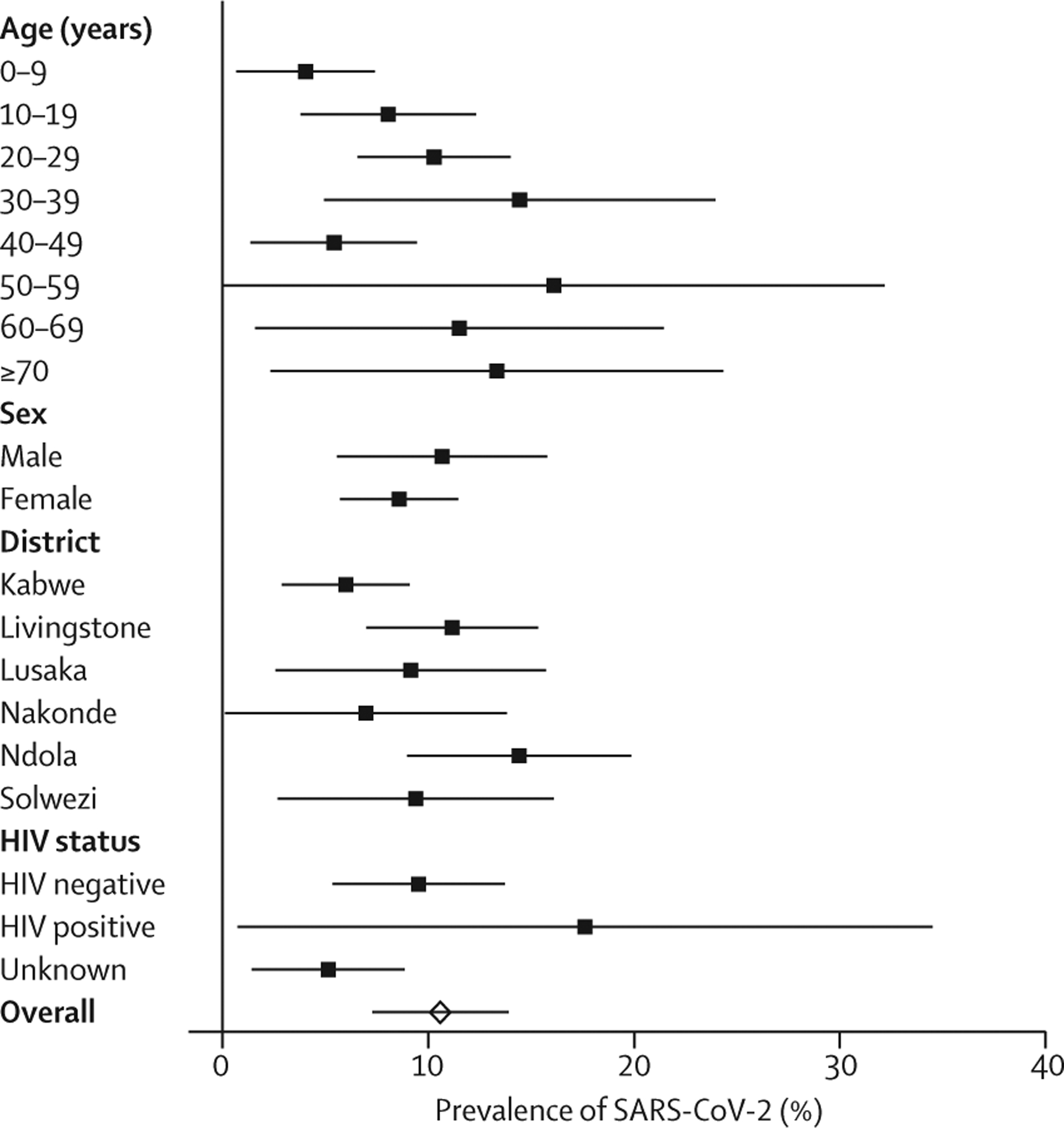
SARS-CoV-2 prevalence for the combined measure by demographic variables in Zambia (July, 2020)

**Table 1:** Participant demographics

	Participants (n=4258)
**Sex**	
Male	49·4% (47·0–51·8)
Female	50·6% (48·2–53·0)
**Age, years**	
0–9	29·3% (25·0–33·6)
10–19	22·3% (20·0–24·7)
20–29	19·4% (17·8–21·1)
30–39	14·2% (11·6–16·7)
40–49	8·3% (5·9–10·6)
≥50	6·5% (5·2–7·9)
**District**	
Kabwe	5·6% (2·1–9·1)
Livingstone	4·8% (1·9–7·8)
Lusaka	63·1% (49·5–76·6)
Nakonde	4·0% (1·5–6·6)
Ndola	14·9% (6·6–23·3)
Solwezi	7·5% (2·9–12·2)
**Location**	
Rural	7·0% (3·8–10·3)
Urban	93·0% (89·7–96·2)
**Nationality**	
Zambian	99·0% (98·3–99·7)
Other	1·0% (0·3–1·7)
**Educational attainment**	
None	16·2% (13·2–19·2)
Primary	40·0% (37·1–43·0)
Secondary	33·9% (31·2–36·6)
Higher	9·4% (7·2–11·7)
Unknown	0·4% (0·0–0·7)
**Occupation**	
Professional, technical, or managerial	3·5% (2·3–4·8)
Clerical, sales, and services	16·5% (13·6–19·4)
Skilled manual	5·0% (3·4–6·6)
Unskilled manual	2·4% (1·1–3·8)
Domestic service	6·4% (5·1–7·6)
Agriculture	3·8% (2·1–5·6)
Other occupation	1·9% (0·9–2·9)
Student	29·6% (25·6–33·6)
Retired	0·6% (0·2–1·0)
Unemployed	29·7% (25·1–34·3)
Unknown	0·6% (0·2–1·0)
**Wealth quartile** *	
1st (lowest)	10·8% (6·2–15·4)
2nd	25·2% (19·1–31·4)
3rd	18·1% (14·4–21·8)
4th (highest)	45·9% (37·5–54·3)
**Medical history**	
Any history of a comorbid medical condition (one or more of the below conditions)	14·5% (12·1–16·8)
Diabetes	0·6% (0·4–0·9)
Cardiac disease	0·4% (0·1–0·7)
Hypertension	3·8% (2·9–4·6)
Asthma	1·4% (0·6–2·2)
Emphysema or chronic obstructive pulmonary disease	0·2% (0·0–0·4)
Chronic kidney disease	0
Cirrhosis or fatty liver	0·1% (0·0–0·2)
Immunocompromised	0·4% (0·2–0·5)
Cancer	0·1% (0·0–0·3)
Pregnant†	6·3% (4·1–8·4)
HIV	5·1% (3·0–7·2)
Tuberculosis	0·2% (0·1–0·3)
Malaria	3·0% (2·0–4·0)
Other chronic medical condition	1·9% (1·1–2·7)
Unknown	16·7% (11·3–22·2)

Data are % (95% CI). Estimates were weighted, thus raw participant numbers were not reported.

* A composite wealth index variable was constructed through confirmatory factor analysis using varimax rotation and orthogonal transformation for the following household-level questions: “Does your household have the following: electricity, television, refrigerator, sofa, clock, fan?”; “Does anyone in your household have a bank account?”; “What is the main material of the floor?”; “What is the main material of the roof?”; “What type of fuel does your household mainly use for cooking?”; possession of a clock or fan in the household and the type of fuel used for cooking were not included in the summary wealth variable because the primary factor loadings were less than 0·5 and cross-loadings were greater than 0·7; all other variables were summed to create the household wealth variable used in analyses.

† Restricted to women aged 15–49 years.

**Table 2:** SARS-CoV-2 prevalence in six districts of Zambia by rtPCR, ELISA, and combined (July, 2020)*

	Frequency, n	Weighted prevalence, % (95% CI)
rtPCR (n=2848†)	230	7·6% (4·7–10·6)
ELISA (n=2614†)	80	2·1% (1·1–3·1)
Combined measure‡ (n=1886†)	205	10·6% (7·3–13·9)

rtPCR=real-time PCR.

* To maintain the 333 test results that were dissociated from the epidemiological data of the participants, prevalence estimates were weighted using the standardised enumeration area testing response rate instead of household testing response rate and age and sex were calibrated at the district level instead of the individual level; the results of a sensitivity analysis excluding these dissociated test results were not significantly different from the main study findings.

† Participants without a standardised enumeration area (rtPCR n=142; ELISA n=90, of which 66 participants had both rtPCR and ELISA) were excluded from this analysis because they could not be incorporated into the survey design.

‡ The combined measure includes the subset of participants who had both PCR and ELISA tests.

**Table 3:** Estimated number of SARS-CoV-2 infections in six districts of Zambia (March–July, 2020)*

	Kabwe	Livingstone	Lusaka	Nakonde	Ndola	Solwezi	Overall
2020 census population projection, n	237 299	190 419	2 731 696	212 070	585 974	332 649	4 290 107
Estimated number of SARS-CoV-2 infections†, n (95% CI)	14 218 (6854–21 581)	21 258 (13 325–29 191)	249 797 (70 404–429 192)	14 778 (264–29 293)	84 459 (52 560–116 359)	31 234 (8940–53 527)	454 708 (312 705–596 713)
Reported cases in official statistics, n	124	21	3521	692	450	109	4917
Ratio of reported cases to estimated infections	1:115	1:1012	1:71	1:21	1:188	1:287	1:92

* Prevalence estimates for the combined measure were applied to individual district populations and the sum of the six districts populations; because each estimate was made independently, the sum of individual district totals does not equal the total for the six districts overall.

† Calculated by applying the prevalence estimate for the subset of participants who had both rtPCR and ELISA tests to the census population projection.

**Table 4:** Associations between demographic and behavioural factors and SARS-CoV-prevalence (combined measure) in six districts of Zambia in July, 2020 (n=1952)

	Prevalence, % (95% CI)	Prevalence ratio (95% CI)
**Sex**		
Male	10·7% (5·6–15·8)	1 (ref)
Female	8·6% (5·7–11·5)	0·98 (0·95–1·02)
**Age, years**		
0–9	4·0% (0·7–7·4)	1 (ref)
10–19	8·1% (3·8–12·3)	1·04 (1·00–1·08)
20–29	10·3% (6·6–14·0)	1·06 (1·02–1·10)
30–39	14·4% (4·9–23·9)	1·10 (1·01–1·20)
40–49	5·4% (1·4–9·4)	1·01 (0·96–1·07)
≥50	14·7% (4·2–25·1)	1·10 (1·00–1·22)
**District**		
Lusaka	9·1% (2·6–15·7)	1 (ref)
Livingstone	11·2% (7·0–15·3)	1·02 (0·95–1·10)
Nakonde	7·0% (0·1–13·8)	0·97 (0·89–1·06)
Ndola	14·4% (9·0–19·9)	1·06 (0·98–1·14)
Kabwe	6·0% (2·9–9·1)	0·97 (0·91–1·04)
Solwezi	9·4% (2·7–16·1)	1·00 (0·93–1·09)
**Location**		
Rural	3·0% (0·0–6·2)	1 (ref
Urban	10·7% (7·1–14·4)	1·08 (1·03–1·13)
**Educational attainment**		
None	1·7% (0·0–3·8)	1 (ref)
Primary	6·0% (3·4–8·6)	1·11 (1·02–1·20)
Secondary	13·9% (7·7–20·1)	1·12 (1·06–1·18)
Higher	12·5% (4·4–20·7)	1·04 (1·01–1·07)
**Any comorbid condition**		
No	9·3% (5·0–13·5)	1 (ref)
Yes	11·4% (4·4–18·5)	1·02 (0·96–1·08)
**Diabetes**		
No	9·7% (5·9–13·4)	1 (ref)
Yes	9·9% (0·0–26·1)	1·00 (0·86–1·17)
**Cardiovascular disease**		
No	9·5% (5·9–13·0)	1 (ref)
Yes	24·6% (0·0–71·0)	1·14 (0·79–1·63)
**Hypertension**		
No	9·6% (5·7–13·5)	1 (ref)
Yes	11·1% (3·9–18·3)	1·01 (0·94–1·09)
**Asthma**		
No	9·9% (6·1–13·7)	1 (ref)
Yes	1·9% (0·0–4·9)	0·93 (0·89–0·97)
**Pregnant** *		
No	9·5% 5 (6·0–12·9)	1 (ref)
Yes	11·7% (0·0–28·5)	1·02 (0·88–1·19)
**HIV**		
Negative	9·7% (5·5–14·0)	1 (ref)
Positive	17·6% (1·5–33·6)	1·07 (0·93–1·23)
**Malaria**		
No	10·1% (6·2–14·0)	1 (ref)
Yes	8·3% (0·0–17·5)	0·98 (0·90–1·07)
**Contact with a laboratory-confirmed COVID-19 case**		
No	10·0% (5·9–14·0)	1 (ref)
Yes	3·3% (0·0–7·3)	0·94 (0·89–0·99)
Don’t know	8·8% (5·0–12·7)	0·99 (0·95–1·03)
**Travel**		
International	7·2% (0·0–22·9)	1 (ref)
Domestic	7·6% (3·6–11·6)	1·00 (0·88–1·15)
None	10·1% (6·1–14·1)	1·03 (0·89–1·19)
**In-person attendance to work or school**		
No	9·7% (5·9–13·5)	1 (ref)
Yes	10·4% (2·8–18·0)	1·01 (0·94–1·08)
**Visited a health facility in the past month**		
No	8·9% (5·6–12·3)	1 (ref)
Yes	12·7% (3·7–21·7)	1·03 (0·96–1·11)
**Number of visits to the market or grocer in the past month**		
0	6·6% (2·4–10·8)	1 (ref)
1–2	8·6% (2·1–15·1)	1·02 (0·95–1·09)
3–5	15·3% (7·2–23·5)	1·08 (1·00–1·18)
5–10	9·1% (4·1–14·0)	1·02 (0·97–1·07)
≥10	8·5% (3·0–13·9)	1·02 (0·97–1·07)
**Usual means of transportation**		
Car	16·4% (1·8–31·1)	1 (ref)
Taxi	8·7% (0·3–17·1)	0·93 (0·81–1·08)
Bike	17·7% (0·0–47·4)	1·01 (0·76–1·34)
Minibus	6·4% (2·2–10·7)	0·91 (0·80–1·05)
Walking	10·7% (5·6–15·7)	0·95 (0·83–1·09)
Don’t know	2·2% (0·0–6·6)	0·88 (0·77–1·00)

Estimates were weighted, thus raw participant numbers were not reported.

* Restricted to women aged 15–49 years.

## Research in context

### Evidence before this study

Since the start of the SARS-CoV-2 outbreak in early 2020, many national and subnational prevalence estimates have been reported around the world. However, few prevalence estimates have been reported for Africa. Although testing has been less widely available in Africa than other parts of the world, the disease burden predicted early in the pandemic on the basis of the experience of other countries has not been observed in the continent. We searched PubMed from database inception to Jan 3, 2021, for peer-reviewed and preprints using the search terms “COVID-19” AND “prevalence” AND “Africa”. Additionally, we searched bibliographies of identified studies, a database of seroprevalence studies maintained by WHO, and the Google search engine for manuscripts and unpublished reports. We identified 11 studies reporting prevalence estimates from seven African countries: Congo (Brazzaville; n=1), Ethiopia (n=2), Kenya (n=1), Malawi (n=1), Nigeria (n=3), South Africa (n=2), and Togo (n=1). All studies were cross-sectional and included varying populations (eg, blood donors, antenatal clinic attendees, health workers, people with HIV), and most had small sample sizes. Most studies used only antibody tests to estimate seroprevalence and none were population-based studies. SARS-CoV-2 prevalence estimates ranged from 1·6% to 45·1%.

### Added value of this study

To our knowledge, this is the first population-based SARS-CoV-2 prevalence study done in Africa. The findings showed high prevalence of rtPCR-positive SARS-CoV-2 infections in Zambia in July, 2020, which was a period of community transmission in the country. Transmission might have been minimal before the first wave in July and August, 2020. Few people who tested positive for SARS-CoV-2 were symptomatic.

### Implications of all the available evidence

This study demonstrates that laboratory-confirmed case counts might be underestimated by an order of magnitude or more in Zambia. The inclusion of rtPCR testing in the study design identified a substantial proportion of people who tested positive for SARS-CoV-2, compared with serological testing alone. The low proportion of symptomatic infections could partly explain the discrepancy between the number of estimated infections and the lower than expected impact of COVID-19 in Zambia. Reasons for the apparent lower COVID-19 severity observed in Africa warrant further study.
