# Supplementary material for: Prevalence of SARS-CoV-2 in six districts in Zambia in July, 2020: a cross-sectional cluster sample survey
Source: Lancet Glob Health. Author manuscript; Available in PMC 2021 Aug 24. (PMC8382844; doi:10.1016/S2214-109X(21)00053-X)
Supplement: Appendix [file NIHMS1733840-supplement-Appendix.pdf]

THE LANCET
Global Health

Supplementary appendix

This appendix formed part of the original submission and has been peer reviewed. We post it as supplied by the authors.

Supplement to: Mulenga LB, Hines JZ, Fwoloshi S, et al. Prevalence of SARS-CoV-2 in six districts in Zambia in July, 2020: a cross-sectional cluster sample survey. *Lancet Glob Health* 2021; published online March 9. https://doi.org/10.1016/S2214-109X(21)00053-X.

***Appendix* - Prevalence of Severe Acute Respiratory Syndrome Coronavirus 2 (SARS-CoV-2) Infection in Six Districts in Zambia—July 2020: a cross-sectional multi-stage cluster-sampled household survey**

Contents

Supplemental Figure 1. Laboratory-confirmed COVID-19 cases by date of confirmation in Zambia, with dates of prevalence study overlaid—Zambia, March-December 2020 2

Supplemental Figure 2. Geographic location and data collection time period of SARS-CoV-2 prevalence survey—Zambia, July 2020 3

Supplemental Figure 3. Flow diagram of sample sizes in the SARS-CoV-2 prevalence study in six districts in Zambia, July 2020 4

Supplemental Table. Symptomology of persons with symptomatic SARS-CoV-2 infections during SARS-CoV-2 prevalence study — Zambia, July 2020 5

Supplemental Figure 1. Laboratory-confirmed COVID-19 cases by date of confirmation in Zambia, with dates of prevalence study overlaid—Zambia, March-December 2020

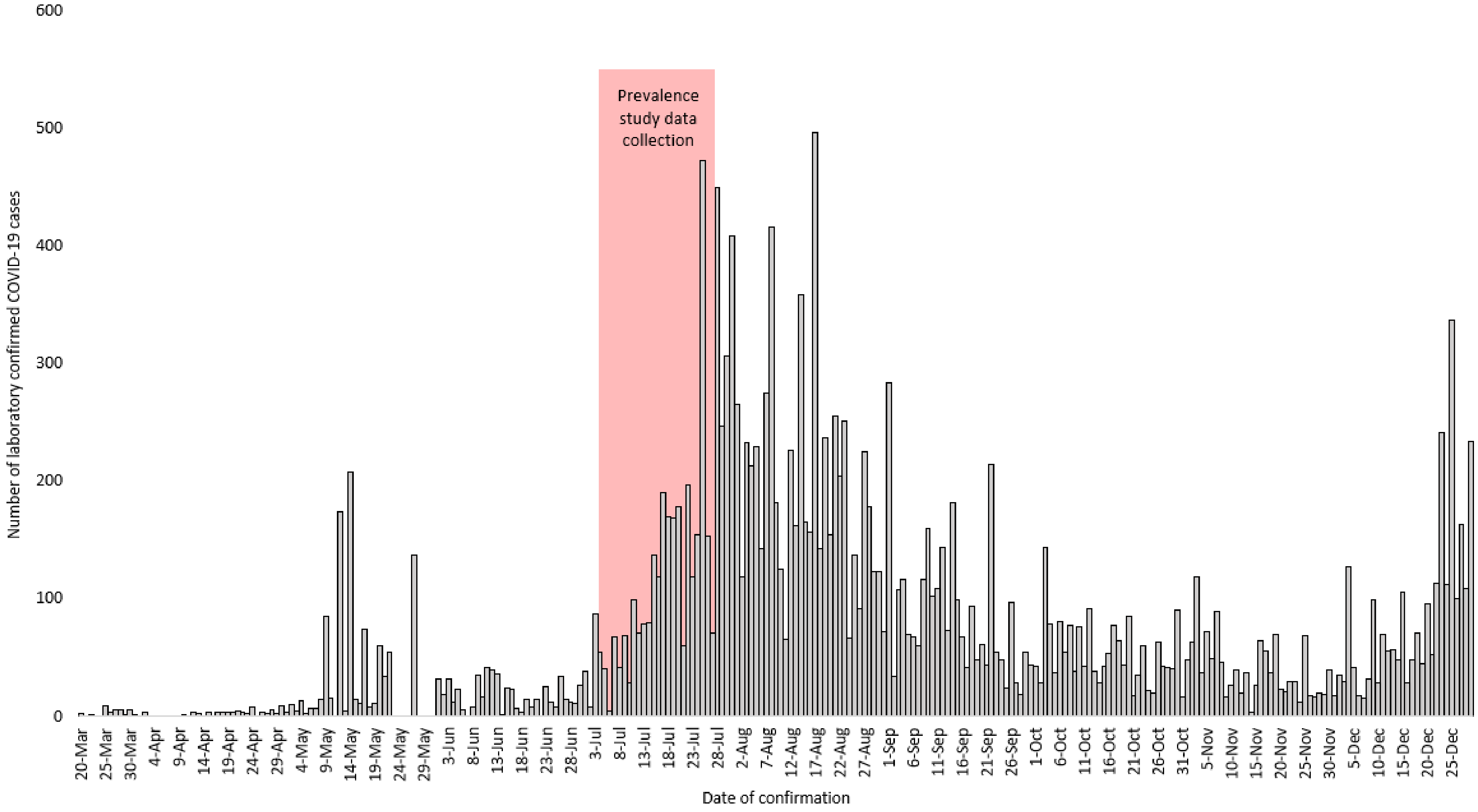

Supplemental Figure 2. Geographic location and data collection time period of SARS-CoV-2 prevalence survey—Zambia, July 2020

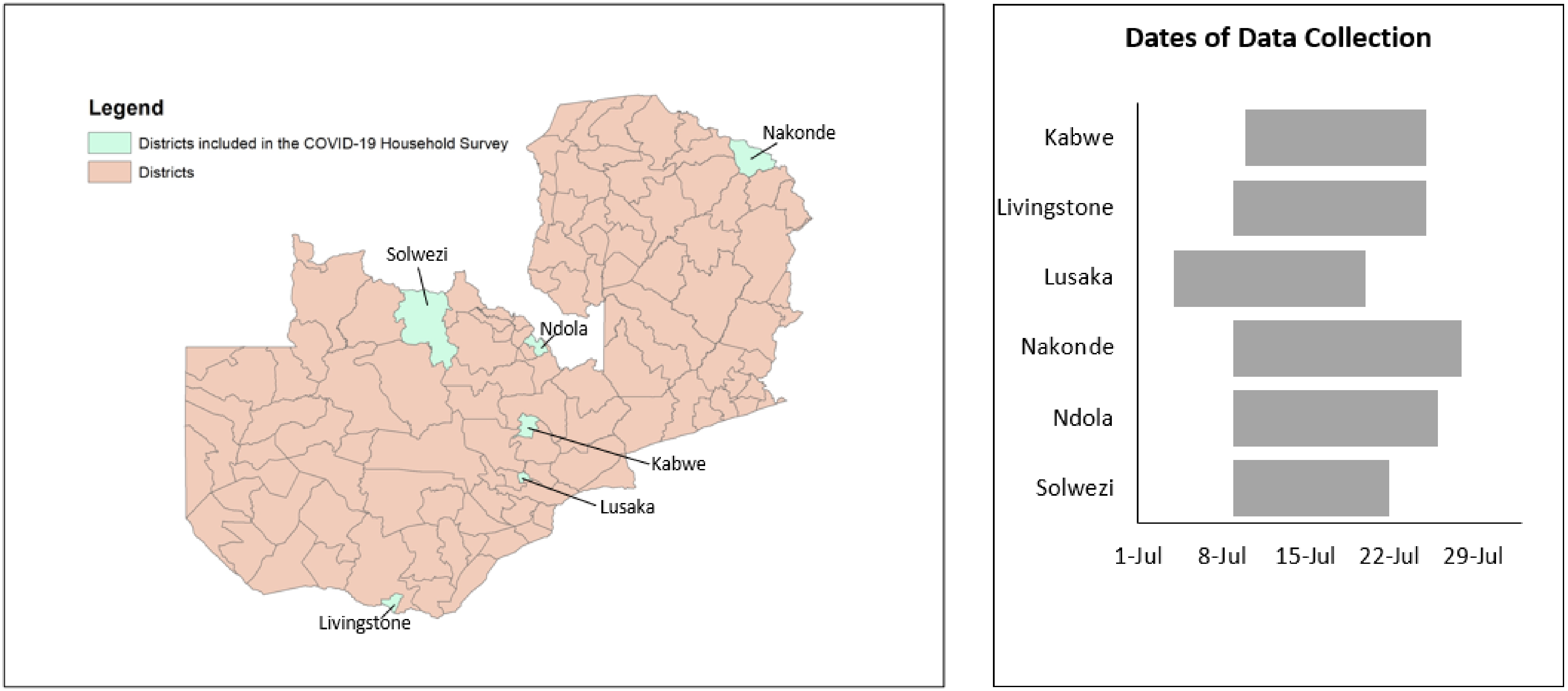

Supplemental Figure 3. Flow diagram of sample sizes in the SARS-CoV-2 prevalence study in six districts in Zambia, July 2020*

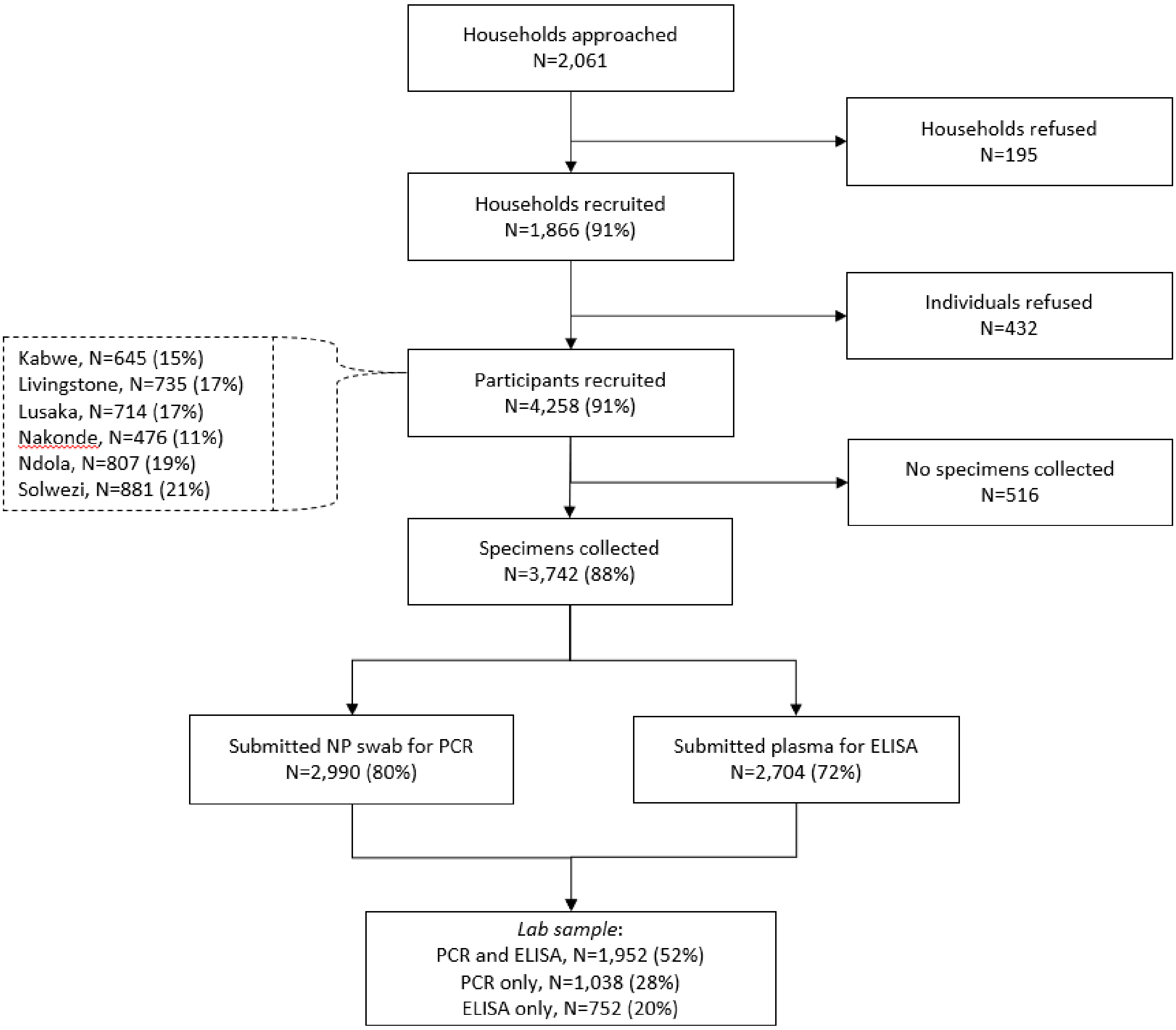

* Lab results for 333 (8.9% of all lab results) participants were disassociated from their epidemiological data. For these participants, their test results data were calibrated at the district-level age and sex distributions

Supplemental Table. Symptomology of persons with symptomatic SARS-CoV-2 infections during SARS-CoV-2 prevalence study — Zambia, July 2020

**Variable**		**% (95% CI)**
Asymptomatic		76.2 (60.0-92.4)
Symptoms (N=35)	Headache	63.6 (13.2-100.0)
	Chills	40.9 (0.9-80.9)
	Cough	25.7 (0.0-69.1)
	Rhinorrhea	21.7 (0.0-62.2)
	Fever	16.0 (1.5-30.5)
	Abdominal pain	5.3 (0.0-12.4)
	Loss of appetite	5.2 (0.0-11.0)
	Nausea or vomiting	5.0 (0.0-11.7)
	Fatigue	4.9 (0.0-10.2)
	Arthralgias	3.6 (0.0-9.0)
	Chest pain	3.0 (0.0-7.6)
	Myalgias	1.3 (0.0-4.3)
	Sore throat	1.3 (0.0-4.3)
	Diarrhea	1.2 (0.0-3.9)
	Decreased taste sensation	0.9 (0.0-2.8)
	Decreased smell sensation	0.9 (0.0-2.8)
	Dyspnea	Not calculated
	Conjunctivitis	Not calculated
	Wheezing	Not calculated
	Other respiratory symptoms	Not calculated
	Nose bleeding	Not calculated
	Rash	Not calculated
	Seizure	Not calculated
	Altered consciousness	Not calculated
	Other neurological symptoms	Not calculated
	Any other symptoms	6.2 (0.0-19.0)
	Don't know	Not calculated
Symptoms required medical attention		65.1 (37.9-92.2)
Symptoms required hospitalization		5.7 (0.0-12.4)
CI: confidence interval		
